# The role of primary cilia in thyroid diseases

**DOI:** 10.3389/fendo.2023.1306550

**Published:** 2024-01-08

**Authors:** Zijiao Tian, Xinlin Li, Xue Yu, Shuxin Yan, Jingwei Sun, Wenxin Ma, Xiaoyun Zhu, Yang Tang

**Affiliations:** ^1^ College of Traditional Chinese Medicine of Beijing University of Chinese Medicine, Beijing, China; ^2^ Guang’anmen Hospital, China Academy of Chinese Medical Sciences, Beijing, China

**Keywords:** primary cilia, thyroid diseases, thyroid cancer, graves’ disease, Hashimoto’s thyroiditis, hypothyroidism, thyroid nodule

## Abstract

Primary cilia (PC) are non-motile and microtube-based organelles protruding from the surface of almost all thyroid follicle cells. They maintain homeostasis in thyrocytes and loss of PC can result in diverse thyroid diseases. The dysfunction of structure and function of PC are found in many patients with common thyroid diseases. The alterations are associated with the cause, development, and recovery of the diseases and are regulated by PC-mediated signals. Restoring normal PC structure and function in thyrocytes is a promising therapeutic strategy to treat thyroid diseases. This review explores the function of PC in normal thyroid glands. It summarizes the pathology caused by PC alterations in thyroid cancer (TC), autoimmune thyroid diseases (AITD), hypothyroidism, and thyroid nodules (TN) to provide comprehensive references for further study.

## 1 Introduction

Primary cilia (PC) are immobile, rod-shaped micro-organelles that extend as a solitary unit from the basal body in most cell types of vertebrates (1–3). Initially, follicular cells with PC were discovered in some embryos and mature vertebrate species (4). PC are the signal center of multiple signaling transmission pathways. Specific lipids and receptors on PC enable them to sense various extracellular chemical and mechanical signals and transmit them to the intracellular level, invoking responses that regulate varying cellular developmental and physiological processes (1, 5). The PC structure consists of a basal body, a transition zone, and an axoneme (6). The basal body defines cell polarity and initiates ciliogenesis. Dysfunctions in ciliogenesis can lead to a variety of ciliopathies (7), which may display clinical phenotypes of congenital hypothyroidism (8). The transition zone connects the basal body and axoneme backbone and acts as a docking medium for intraflagellar transport (IFT) particles, which are responsible for transporting all proteins into and along the ciliary compartment (9). Proteins are transported along PC from the base to the tip by anterograde IFT, which is catalyzed by cytoplasmic dynein 2/1b motor, and from the tip to the base of PC by IFT catalyzed by kinesin-2 motor (10). Alpha and beta tubulin form the axoneme, a microtubule structure modified post-translationally to avoid depolymerization. PC length ranges from 5.0 mm to 10.7 mm and has a mean length of 7.3 ± 1.2 mm (4). PC length is associated with cilia-mediated signaling function and altered by agonists for receptors (11), transcriptional regulation of IFT genes, and actin depolymerization (12). Defects in PC length and morphology can lead to dysregulation of signaling transduction and cellular functionality, which contribute to developing diseases termed ciliopathies (13). An experiment showed that the frequency of PC in normal human thyroid follicles is 67.53 ± 3.62% (5). Considering the potential of concentrating signal cascade proteins within ciliary lumens and membranes, PC are an ideal mediator for regulating endocrine pathways (14).

The mammalian thyroid gland regulates the synthesis and secretion of thyroid hormone (TH). TH plays a crucial role in normal development, growth, neural differentiation, and metabolic regulation in mammals (15–17). Furthermore, it can regulate adult hippocampal neurogenesis, which is responsible for learning, memory, and mood by acting through thyroid hormone receptorα (TRα) (18, 19). As the basic unit of the thyroid gland, globular-shaped, vascular, encircled, and colloid-filled follicles are essential for its functional integrity. The colloid-facing membrane of follicular epithelial cells functions as an active exchange interface in hormone production (5), and follicular cells are a prerequisite for TH synthesis (20). Thyroid cancer (TC), Graves’ disease (GD), Hashimoto’s thyroiditis (HT), hypothyroidism, and thyroid nodules (TN) are common thyroid diseases worldwide. Studies show that thyroid illnesses emerge due to oncogenic mutations and abnormal changes in some intracellular downstream signaling pathways and cytokines (21–23). Previous research revealed that as a key mediator, PC influences the pathogenesis of thyroid abnormalities in function and structure (6). However, there are no inductive studies that describe the systematic role of PC in thyroid diseases.

The purpose of this article is to reveal the role that PC in follicular epithelial play in thyroid diseases and provide novel ideas for further research.

## 2 Primary cilia and thyroid cancer

Thyroid cancer is one of the endocrine cancers with the highest morbidity and a rapidly and steadily rising incidence (24).

In patients with TC, the length and frequency of PC change when responding to extracellular and intracellular stimuli (6). However, there are differences in the length and frequency changes in PC among different TC phenotypes. A previous study found that compared to individuals with normal thyroid glands, patients with follicular thyroid cancer (FTC) and papillary thyroid cancer (PTC) display PC with significantly increased length and invariable frequency, and oncocytic PTC variants exhibit PC with decreased length and frequency (25). PC frequency is observably lower in anaplastic thyroid carcinoma (ATC) (5) and is associated with TC tumorigenesis and TC progression (26). Researchers believe that the absence of PC in PTCs leads to increased apoptosis and is associated with reduced tumor aggressiveness and malignant potential (16).

A mouse model with PC loss mediated by intraflagellar transport 88 (IFT88) showed an irregularly dilated thyroid gland with destroyed follicles, malignant properties, and progressively differentiated thyroid cancer (PDTC) (5), which reflected the pathogenicity of PC mutations in the thyroid gland. As mentioned in the literature review, these findings confirm that abnormal changes in PC structure are closely related to TC tumorigenesis and progression. Thus, maintaining normal ciliogenesis is probably a therapeutic target for TC.

As the mediator of signaling pathways, PC play an essential role in regulating the development of various subtypes of TC. Communication and interflow between tumor microenvironments and TC cells are affected by alterations in PC and influence the prognosis and therapeutic effect on TC (27–30).

IFT88 plays a critical role in anterograde transport in ciliary proteins (31, 32), which maintains bidirectional motility along the axonemes and is indispensable for ciliogenesis and functional proficiency (33). Therefore, dysfunctional mutations of IFT88 lead to severe defects in ciliogenesis (26). Many studies have examined IFT88 function and demonstrated PC loss in many types of cancer (34–37) associated with higher cancer aggressiveness (5, 38, 39). A previous experiment showed that thyroid-specific IFT88-deficient mice developed TC without additional activation of thyroid oncogenic kinases. Clinical studies have provided evidence that IFT88/PC dysfunction causes abnormalities in cellular metabolism, such as oxidative phosphorylation (OxPhos), decreased mitochondrial membrane potential, reduced ATP synthesis, and increased aerobic glycolysis with increased fatty acid synthesis, loss of mitochondrial function, and even mitochondrial fragmentation in rare cases (26). The mechanism of TC resulting from loss of function (LOF) of IFT88/PC is inconsistent in different studies. LOF of IFT88 prevents the PC-mediated Hedgehog pathway from being used to promote carcinogenesis caused by SmoM2. Nevertheless, lesions resembling basal cell carcinoma can be accelerated by LOF of IFT88 through a tumorigenic pathway independent of the PC (34). Therefore, the specific role of LOF of IFT88/PC in TC needs further exploration.

The Hedgehog (Hh) pathway is crucial for vertebrate embryonic development, and misregulation is responsible for many cancers (40). Studies have reported abnormal Hedgehog (Hh) pathways activated by PC (41). Sonic Hh (Shh) (one of the secreted proteins) belongs to the Hedgehog family (42). Immunolocalization tests demonstrate that scores of core components of Sonic hedgehog (Shh) signal transformation localize on PC, and PC is fundamentally important for canonical Shh signaling in vertebrates (29). Shh ligand stimulates Gli (including Gli1, Gli2, and Gli3) transport from PC to the nucleus, where they activate Hh target genes (41). Hh pathway is considered a bona fide ciliary pathway presently (1). PC mediate the interaction between stroma and cancer cells, the defects of which may interfere with the interaction that mediates the aberrant activation of Hh pathways. This finding proves that the Hh pathways mediated by PC are likely to affect TC tumorigenesis (6). In the early stage of TC development, PC-mediated growth factor binding to RTKs triggers the activation of the MAPK and PI3K-AKT cascades, which regulate TC cell proliferation. Increased RTK activity promotes RET/RAS/BRAF mutations (43, 44), which influence Hh pathway activation without ligands in tumor cells (45), implying that PC carry the signaling proteins above. Hh and RTK signaling crosstalk are integrated by PC, which coordinate the synthesis and development of TH. Therefore, PC mutations can influence pathways related to tumorigenesis and the development of TC directly.

NIMA-related kinases (Nek), LKB1, Aurora kinase A (AURKA), and polo-like kinase (Plk1) are PC-related proteins identified as crucial for PC regulation.

Situated in PC and centrosomes, Nek may help to coordinate cell cycle progression and ciliogenesis (46). Studies have shown that cells lacking NIMA or some NIMA-related kinases suffer from chromosome segregation and mitotic errors and then undergo apoptosis (47), manifesting as bizarre and heterogeneous PC (48). In both the classical and follicular variants of PTC, overexpression of Nek1 is frequently associated with aggressiveness, which is highly specific and sensitive. Therefore, Nek 1 may affect the identification of malignant features during TC diagnosis (49).

The LKB1, a tumor suppressor kinase located in PC on epithelial cells, is known to inhibit mTOR activation by inhibiting AMPK signaling, which represses tumor cell polarization and metastasis (47, 50). Therefore, LKB1 expression negatively correlates with increased tumor aggressiveness and is assumed as a prospective therapeutic target for TC (51, 52).

AURKA is located in the basal body, centrosome, and radial microtubules of PC, participates in cellular responsiveness to growth factors, and regulates ciliary disassembly (53, 54). AURKA gene amplification or overexpression is linked to malignancies, such as colon, liver, pancreatic, breast, and gastric cancers (39, 55). Previous studies suggested that AURKA could physiologically cause lung cancer and breast cancer through weakening LKB1/AMPK signaling pathways and mediating resistance to autophagic cell death, respectively (56, 57). Research reveals that the genes encoding the Aurora kinases induce malignant transformation of thyrocytes, and some TC-derived cell lines and tissues exhibit the detection of their overexpression, implying a poor prognosis (6). Although the evidence that AURKA influences TC is insufficient, it can be inferred from the mechanism of diverse cancers. Thus, it is probably a novel and valuable therapeutic target of TC at present (58–60). In preclinical research and clinical trials, MLN8237, a conformation-changing AURKA inhibitor, demonstrated exceptional anticancer activity by inhibiting AURKA (61).

Considered a cellular proliferation marker, Plk1 is localized in the PC transition zone in epithelial cells (62). The small-molecule inhibitor of Plk1 activity can limit the first two phases of ciliary disassembly, while induced PC disassembly can evoke the activity of Plk1 kinase (63). Therefore, this interrelationship between Plk1 and ciliary disassembly can serve as one of the arguments that Plk1 is a PC-related protein critical for PC regulation (6). The findings of several studies show that PLK1 is unlikely to contribute directly to the mitosis of papillary carcinoma cells. One of the explanations is that PLK1 plays an oncogenic role and is constitutively required in papillary carcinoma during the early phase, while it is less necessary for the development of this carcinoma during the advanced stage (64–66). Plk1 is only expressed occasionally in normal thyrocytes while overexpressed in the bulk of microcarcinomas, smaller PTC, ATCs, and incidental carcinomas, which may support the assumption (67) that Plk1 makes a constitutive effect on PTC in the early stage. Furthermore, several tests related to PLK1 inhibitors show promising results in preclinical settings (68–71). Yet, clinical investigations reveal that hematologic toxicity (neutropenia) frequently causes therapeutic action to occur at or beyond the maximum tolerated dosage (72–74).

Cysteine cathepsins are a group of proteases that are proteolytically active to different extents (75). Secreted from Nthy-ori 3-1 cells *in vitro* (76) and from human thyrocytes *in situ* (77), cysteine cathepsins contribute to tissue homeostasis in the thyroid gland (78). Cysteine cathepsins B, K, L, and S are involved in the proteolytic processing and degradation of thyroglobulin (Tg) in the follicular lumen for initial TH liberation (77, 79). The production of prohormone Tg and its proteolytic processing are essential for thyroid function. A previous study by Alara Gaye Doğru et al. discovered the presence of cysteine cathepsins B and L at the PC of Fisher rat thyroid (FRT) cells, indicating their localization, and showed that inhibiting these cysteine cathepsins leads to the elimination of the PC (80). An interesting possibility is that different substrates of truncated cathepsin B and V variants are present in the nuclei of thyroid carcinoma cells (75). Several studies implicate the involvement of cysteine cathepsins in malignancies and cancer progression due to an increased expression and activity in cancer cells and tumor-associated tissue. As deduced from co-localization studies and *in vitro* degradation assays, a study suggested that nuclear variants of cathepsins are involved in the development of thyroid malignancies (75).

Thyroid-stimulating hormone (TSH) receptor activation increases cytosolic calcium levels, stimulating the release of cysteine cathepsins. These cysteine cathepsins then enzymatically break down Tg to release TH. The ultimate result of Tg processing is the production of Tg fragments that function as thyropins, which effectively suppress the activity of cysteine cathepsins (81). This process affects thyroid autoregulation and contributes to thyroid diseases.

Although the mechanisms of PC loss in TC cells remain uncertain, the survival of TC cells is determined by proper regulation of PC proteins between ciliogenesis and the cell cycle (82). Considering these PC proteins as targets for treating TC is a relatively new idea.

Studies on the drug effects on PC in human cells are limited. Notably, specific drugs, for example, U0126 and ganetespib (83, 84), affect disassembling PC, restoring ciliogenesis, shortening PC, preventing Smo accumulation in PC, and inhibiting the proliferation of TC cells in different histological types (6, 85, 86). Many drugs are shown to be effective in TC treatment. For example, docetaxel lowers PC levels in olfactory cells, paclitaxel produces PC elongation in the quail oviduct, doxorubicin increases PC synthesis in breast fibroblasts (87), and carboplatin causes PC disassembly in sensory cells (88). Several RTK inhibitors, such as sorafenib, lenvatinib, vandetanib, and cabozantinib, were approved for clinical practice for treating differentiated thyroid cancer (DTC), PDTC, and metastatic thyroid cancer (MTC) (89). However, there is no information on the effects of RTK inhibitors on PC. They have been evaluated in preclinical and clinical investigations in TC, and taking these aspects into account will help develop logical therapy methods for TC. Therefore, restoring PC in TC may be a promising therapeutic strategy (90). Thus, there is a broad scientific prospect of developing drugs with PC as a target for thyroid cancer therapy.

## 3 Primary cilia and autoimmune thyroid disease

As the most common autoimmune disease, autoimmune thyroid disease (AITD) is prevalent in approximately 5% of the population (91, 92). GD and HT are two main clinical presentations of AITD, both characterized by lymphocytic infiltration of the thyroid parenchyma (93). The pathological process of GD is influenced by a defective Taar1 located in the PC. The shedding of PC on kidney epithelial cells indicates that oxidative stress may damage PC in thyroid epithelial cells, while the pathological process of HT is regulated by PC, which mediate apoptosis and affect the normal expression of miRNA. A summary of past research confirms that PC play an essential role in AITD, especially GD and HT.

### 3.1 Primary cilia and Graves’ disease

Thyrotoxicosis is an autoimmune response characterized by the presence of autoantibodies targeting the TSH receptor (TSHR-Ab), resulting in goiter and hyperthyroidism (93, 94). GD is one of the most common causes of thyrotoxicosis. Moreover, the thyroid gland of GD is infiltrated by autoreactive lymphocytes and circulating thyroid antibodies (23).

Although the precise pathogenic mechanisms remain unknown, researchers have revealed a close correlation between morbidity and sex and susceptibility genes (95, 96). Most current research shows an increase in the length and frequency of PC, whereas studies exploring the mechanisms underlying the influence of PC on GD are rare. Therefore, the central links (Taar1, oxidative stress) that may be related to PC in GD pathogenesis are summarized here to fill the gap in this field.

Recent research shows that GD results from structural and functional defects in PC, also known as ciliopathies (9, 97, 98). Defects in ciliogenesis should appear in GD, characterized by follicle integrity changes, deregulation of hormone synthesis, and altered proliferation rate (99). Until now, the role of PC has not been demonstrated directly in the thyroid, while it can be inferred from existing work. In previous experiments, GD follicular cells were characterized by a convex surface adorned with microvilli, and small cilia that resembled microvilli were infrequently seen near the cell core (23). In follicular cells of GD, PC length and frequency decreased significantly compared to the normal group, and the absence of PC intensified the alterable presence of follicles in apoptosis (99). The findings proved that ciliogenesis plays a key role in sustaining the normal physical condition of follicular cells and directly impacts the functional abnormalities of the thyroid gland (6, 99). Moreover, GD thyroid samples with shorter axonemal and lower ciliary frequencies had a worse response to antithyroid medication therapy (99). Therefore, the therapeutic effect may be enhanced by maintaining the normal length and frequency of the PC, providing a novel idea in clinical research and treatment.

The hypothalamic–pituitary–thyroid (HPT) axis is a vital regulatory pathway of the thyroid gland, in which low concentrations of TH trigger negative feedback, resulting in the release of thyrotropin-releasing hormone (TRH) from the hypothalamus and TSH from the pituitary gland (100–102). Evidence suggests that Taar1, a putative receptor of thyronamines localized at PC (103), is vital to maintaining canonical regulation of the HPT axis (100) and might contribute to hyperthyroidism through its effect on the HPT axis. An experiment revealed that the serum of male mice with Taar1 knocked out showed mild TSH receptor resistance and elevated TSH concentrations (100). Inversely, serum TSH concentrations revealed a decline in serum TSH concentrations in Taar1 knockout male mice with older age, while the increase in serum TSH concentrations was significant when comparing young adult Taar1 knockout male mice (100). These varying results might be attributable to the heterogeneity of the thyroid tissue. That is, Taar1-deficient mice are hyperthyrotropinemic, which is the characteristic of GD. Therefore, there is a high probability that the defect of Taar1 caused by alterations in PC structure and function is related to the pathogenesis of GD.

Moreover, there is evidence that oxidative processes play a role in GD pathogenesis (104). Abalovich et al. (105) found that hyperthyroid and GD patients showed an increase in oxidative stress markers and a decrease in markers of the antioxidant system. Previous studies have also revealed that hyperthyroid patients demonstrate elevated levels of oxidative stress markers in plasma and other tissues (106–108). Presently, there is insufficient evidence on the effect of oxidative stress on PC, hence eventually causing GD. Studies on ischemia/reperfusion injury have shown that, to some degree, excessive ROS production and oxidative stress lead to the shedding of PC on kidney epithelial cells (67), implying that oxidative stress may damage PC in thyroid epithelial cells as well and contribute to thyroid disease pathogenesis accordingly.

### 3.2 Primary cilia and Hashimoto’s thyroiditis

Autoimmune thyroiditis, or HT, is a chronic inflammatory autoimmune illness that uses the thyroid tissue as an antigen. The incidence has increased considerably in recent years (109, 110). Autoimmune thyroiditis is a cellular autoimmune illness with apparent inflammatory infiltration that destroys the thyroid gland (23). In children and teenagers, it is the most prevalent cause of goiter and acquired hypothyroidism.

Researchers proved that the frequency and length of PC decreased significantly in experimental specimens compared to controls. Follicular cells in HT exhibited a variety of forms and uneven borders, which occasionally created difficulty in distinguishing between different cells. Each cell core was invaded by hypomorphic cilia, which occasionally resembled “volcano-like” formations (23).

The loss of thyroid epithelial cells is a hallmark of HT. An experiment revealed that the percentage of *in situ* apoptotic thyrocytes increases in HT; thus, the apoptosis of thyroid follicular cells plays a vital role in the pathogenesis of HT (111). Apoptosis can be initiated in various ways and affects several cellular processes (112). Junguee Lee et al. believed that HT is usually associated with mitochondrial dysfunction (25). As crucial regulators of cell death, mitochondria act by an intrinsic process called the mitochondria-dependent pathway of apoptosis. The intrinsic apoptotic route is normally mediated by mitochondrial outer membrane permeabilization (MOMP), which includes the voltage-dependent anion channel (VDAC). Moreover, VDACs concentrate in the basal body of PC, among which VDAC1 and VDAC3 regulate ciliogenesis negatively (113). A study found that mice lacking PC in thyroid follicular cells showed apoptotic cell death, resulting in altered follicular structure. Furthermore, inhibiting ciliogenesis in thyroid cancer cell lines resulted in VDAC1 oligomerization following VDAC1 overexpression, leading ultimately to apoptosis. Consequently, the LOF of PC is probably one of the potential factors that stimulate apoptogenesis (114).

To summarize, HT samples showed loss of thyroid epithelial cells caused by mitochondrial dysfunction. Furthermore, defects in the structure and function of PC affect mitochondrial dysfunction and apoptosis accordingly. PC, a core link in the entire process, is closely related to HT. Although no relevant reports show that HT may be affected by apoptosis caused by PC abnormality, it is a likely potential mechanism that needs investigation.

As the microenvironment of AITD is clearly pro-inflammatory, cytokines and chemokines are considered important in its pathogenesis (109, 115). As a marker of AITD, thyroid peroxidase antibodies (ATPO) are present in nearly all HT patients (115), which may trigger the synthesis of autoantibodies by MHC expression on thyrocytes and lymphocyte infiltration (116). Especially in HT, cytokines stimulated by T and B lymphocytes enhance the inflammatory response and production of antibodies, which results in thyroid tissue damage by apoptosis (117). Experiments reveal that pro-inflammatory cytokines IFN-γ and TNF-a increase the expression of the genes mentioned above while decreasing the number of PC (23). Previous studies have shown that IL-1 causes cilia to elongate through a mechanism dependent on protein kinase A, indicating the significance of inflammation in controlling PC shape and function (118). Studies validated a decrease in miR-141 expression and associated this decrease with the transforming growth factor beta 1 (TGF-β1) signaling pathway in HT (119); furthermore, they showed that decreased expression of miR146b, miR-221, and miR-222 could play a role in the development of papillary thyroid carcinoma (120). A proposed model of the mechanisms underlying ciliary defects in AITD showed abnormal antigen presentation in the thyroid and breakdown of tolerance to self-antigens, leading to complex and interrelated intrathyroidal immune processes. A combination of a pro-inflammatory environment and increased miRNA expression may repress the expression of ciliary-related genes, leading to impaired ciliogenesis (23). Moreover, miRNA transfection affects ciliary growth in thyrocytes. More critically, studies show that aberrant miRNA expression is the foundation of immune cell differentiation and activation (121), and the regulation between cytokine activities and miRNA expression is bidirectional (122, 123). Moreover, the potential utilization of this pathway as a novel therapeutic approach to treat these disorders arises from the involvement of pro-inflammatory cytokines and miRNA targeting in the formation of PC in thyroid cells (23).

Epithelial–mesenchymal transition (EMT) is a process that occurs under both physiological and pathological conditions. It is characterized by the loss of epithelial characteristics and the acquisition of mesenchymal features by epithelial cells (124). Recent research shows that PC deficiency triggers EMT under resting conditions and exacerbates it under the influence of fibrotic signals such as TGF-β (125, 126). Research data indicate an increase in the acquisition of mesenchymal markers by thyroid follicle cells in AITD that may contribute to the pathogenesis of these diseases. Furthermore, EMT induction by TGF-β in thyroid cells suggests the potential usefulness of this pathway as a novel therapeutic strategy to treat AITD (22).

## 4 Primary cilia and hypothyroidism

Hypothyroidism, which may be acquired or congenital, is related to elevated serum TSH levels and low serum free T4 and/or free T3 levels (127). The prevalence of hypothyroidism rises with age and is higher in women, patients with other autoimmune diseases, and those with Down syndrome and Turner syndrome (128). As an essential micro-organelle mediating signaling pathways and maintaining thyroid homeostasis, PC are speculated to be related to the pathological mechanisms underlying hypothyroidism. However, the association has not received the attention of researchers. The probable mechanism by which PC influence hypothyroidism via protein and hormone levels is discussed below.

Recent research indicates that PC play a significant role in regulating the processing and Gli protein function (129). Presently, the Hh pathway is considered a *bona fide* ciliary pathway, and PC mediate the interaction between stroma and cancer cells, the defects of which may interfere with the interaction that mediates the aberrant activation of Hh pathways. Gli3 is stimulated by the Shh ligand for transport from PC to the nucleus, where Hh target genes are activated. Therefore, Glis3 may be part of the signal transduction pathway mediated by PC, which requires activation before translocation to the cytoplasm and nucleus (130). Known as an anterograde IFT motor, KIF3a is necessary for Gli3 activator formation and proteolysis (131). Glis3 expression is restricted to thyroid follicular cells. A previous study clarified that TH biosynthesis depends on the expression of a specific group of genes directly regulated by Glis3, particularly the two iodide transporter genes, NIS and PDS (132). In Glis3 mutant mice, TSH were found increased, while TH were observed decreased (133), and nkx2.4b and pax2a expression, which are important transcription factors regulating thyroid development, exhibited a decrease in Glis3-deficient zebrafish (134). Humans and mice with loss of Glis3 develop congenital or neonatal hypothyroidism and have a reduced life span (8, 135, 136). Recent studies have identified a link between Glis3 missense mutations and thyroid dysgenesis, as well as the association between the Glis3 variation, rs1571583, and thyroid malfunction regulation (133). Several findings revealed that thyroid dyshormonogenesis, rather than thyroid dysgenesis, was responsible for hypothyroidism in animals lacking Glis3 (137). Although the present research on the mechanism underlying the defects in PC that influence thyroid function and cause hypothyroidism is limited, it can be inferred that PC interact with Glis3 via the signaling pathway and result in thyroid dysfunction.

The ciliary pocket located on PC is an invagination of cytoplasm, where the receptor-mediated endocytosis takes place (138). Tg endocytosis, which has a critical effect on TH release, is primarily mediated by low-density lipoprotein receptor protein 2 (LRP2)/megalin situated at PC and regulated by TSH (139–141). Tg uptake triggered by TSH is a key factor of TH and Tg release into the blood circulation (142–144). Hypothyroidism in megalin knockout mice is accompanied by reduced free T4 (fT4) and serum Tg levels and noticeably higher serum TSH levels (145). A study showed that defective PC cause a significant loss of LRP2/megalin with high TSH levels and colloid Tg depletion (5). Consequently, damaged PC may affect the normal function of LRP2/megalin and result in Tg abnormality, causing hypothyroidism.

There has been no research on the length and frequency alterations in PC. Thus, restoring normal PC formation and shape is probably a promising treatment strategy for hypothyroidism, which requires further study.

## 5 Primary cilia and thyroid nodule

TN is a common clinical disease with one or more structurally abnormal masses in the thyroid gland. It is usually benign (146), with palpable nodules in 5% of the population, especially older patients (147, 148); 5%–15% is proved malignant (149, 150), while the data increase to 35% by positron emission tomography scanning (151).

Thyroid follicular cells are the primary source of TN (152). The follicles in nodular hyperplastic thyroid tissue are highly heterogeneous in size and morphology, ranging from small follicles with little colloid and high columnar thyrocyte linings to very large follicles with abundant colloid and flat epithelium, which probably is a typical pattern of nodular hyperplasia (153, 154).

Fernández-Santos elucidated that trace amine-associated receptor 1 (Taar1), the putative receptor of thyronamines, is located in PC on thyroid follicles and might play a role in regulating cathepsin-mediated proteolysis of Tg and, consequently, TH synthesis (100). They found that, in TN cells, the average PC length and frequency in follicular cells decreased considerably. Although follicles had noticeable size disparities, there was no relation between follicular size and PC mutations (99).

The varied follicular patterns also showed differences in ciliogenesis, with a decreased PC frequency in areas with changed follicles compared to those with predominant normal-appearance follicles. In murine thyroid follicles, LOF of PC causes aberrant and irregular follicles that finally give rise to papillary and solid proliferative nodules (5).

We acknowledge the various discussions on the relationship and interactions between PC and TN; however, researchers have still not verified this hypothesis due to insufficient experimental and clinical evidence to support the conjecture at present. Therefore, the relationship between TN and PC needs further investigation.

## 6 Conclusions and prospect

**Figure 1:**
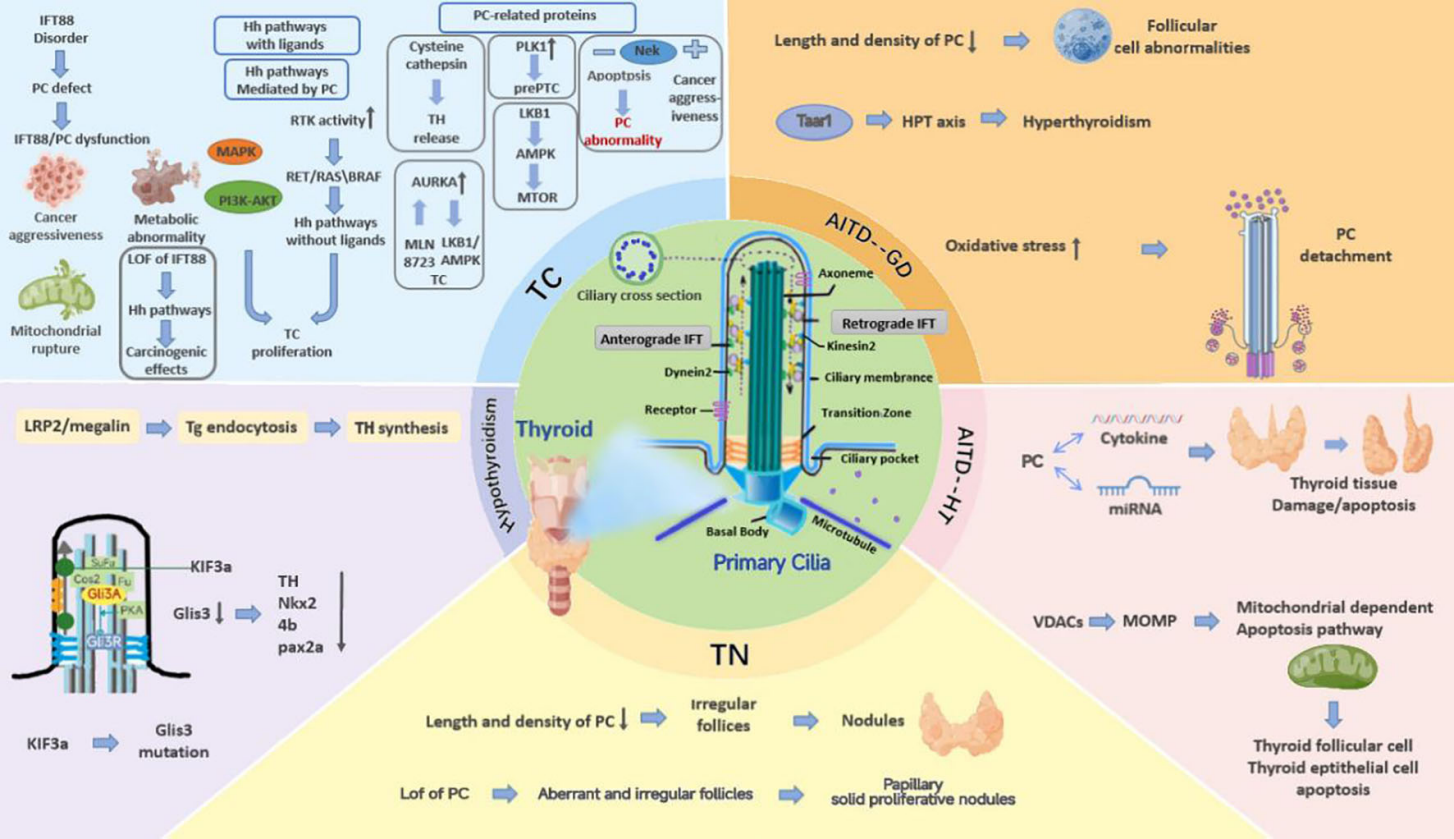
The mechanisms of PC mutations that cause thyroid diseases. PC play an essential role in mediating signaling pathways and sustaining thyrocyte homeostasis in various thyroid diseases. Impaired function and structure of PC affect pathways and proteins associated with TC aggressiveness, and it mediates GD by impairing Taar1 and HT by disrupting apoptosis and affecting normal miRNA expression. Defective PC also result in abnormal secretion of TSH and TH, causing hypothyroidism. Further studies are necessary due to insufficient experimental and clinical evidence on the relationship between TN and PC.

Based on the exposition described above, PC play an essential role in mediating signaling pathways and sustaining thyrocyte homeostasis in various thyroid diseases. Impaired function and structure of PC affect pathways and proteins associated with TC aggressiveness. Furthermore, it mediates GD by impairing Taar1 and HT by disrupting apoptosis and affecting normal miRNA expression. Defective PC also result in abnormal secretion of TSH and TH, causing hypothyroidism. Further studies are necessary due to insufficient experimental and clinical evidence on the relationship between TN and PC. Thus, there is a strong possibility that PC is a potential target in thyroid diseases, and studies on the probable effects of impaired functionality and structure of PC on proteins, signaling pathways, and genes may reveal the mechanisms underlying thyroid disease pathogenesis in the future (**Figure 1**). Restoring PC is probably a promising therapeutic strategy in clinical settings. To assess the role of PC in the incidence and progression of thyroid disorders, preclinical and clinical research is necessary. Thus, further studies are required to explore the pathological mechanism underlying impaired function and structure of PC, provide new research directions for thyroid disease treatment, and develop a broad idea for other diseases related to PC.

## Author contributions

ZT: Writing – original draft, Writing – review & editing. XL: Writing – original draft, Writing – review & editing. XY: Writing – original draft, Writing – review & editing. SY: Writing – review & editing. JS: Writing – review & editing. WM: Writing – review & editing. XZ: Supervision, Writing – review & editing. YT: Funding acquisition, Supervision, Writing – review & editing.
